# The crystal structure of the zwitterionic co-crystal of 2,4-di­chloro-6-{[(3-hy­droxy­prop­yl)azaniumyl]­meth­yl}phenolate and 2,4-di­chloro­phenol

**DOI:** 10.1107/S2056989019012544

**Published:** 2019-09-10

**Authors:** Bhawna Uprety, Charmaine Arderne

**Affiliations:** a Department of Chemistry, University of Johannesburg, PO Box 524, Auckland Park, Johannesburg, 2006, South Africa

**Keywords:** crystal structure, zwitterionic co-crystal, 2,4-di­chloro­phenol, bifurcated hydrogen bonding, π–π inter­actions

## Abstract

The title compound, C_10_H_13_Cl_2_NO_2_·C_6_H_4_Cl_2_O, was formed from the incomplete Mannich condensation reaction of 3-amino­propan-1-ol, formaldehyde and 2,4-di­chloro­phenol in methanol. This resulted in the formation of a co-crystal of the zwitterionic Mannich base, 2,4-di­chloro-6-{[(3-hy­droxy­prop­yl)azaniumyl]­meth­yl}phenolate and the unreacted 2,4-di­chloro­phenol. The compound crystallizes in the monoclinic crystal system (in space group *Cc*) and the asymmetric unit contains a mol­ecule each of the 2,4-di­chloro­phenol and 2,4-di­chloro-6-{[(3-hy­droxy­prop­yl)azaniumyl]­meth­yl}phenolate. Examination of the crystal structure shows that the two components are clearly linked together by hydrogen bonds. The packing patterns are most inter­esting along the *b* and the *c* axes, where the co-crystal in the unit cell packs in a manner that shows alternating aromatic di­chloro­phenol fragments and polar hydrogen-bonded channels. The 2,4-di­chloro­phenol rings stack on top of one another, and these are held together by π–π inter­actions. The crystal studied was refined as an inversion twin.

## Abstract 2

The title compound was isolated serendipitously as the co-crystal of 2,4-di­chloro­phenol and 2,4-di­chloro-6-{[(3-hy­droxy­prop­yl)azaniumyl]­meth­yl}phenolate in its zwitterionic form, from an incomplete Mannich condensation. The co-crystal is held together by extensive intra- and inter­molecular hydrogen bonds as well as π–π inter­actions.

## Chemical context   

The Mannich condensation is an important reaction in synthetic organic chemistry. The formation of the C—N bond in the resulting Mannich base is often an important step in the biosynthesis of several natural products, such as alkaloids and flavanoids (Sarhan *et al.*, 2006 ▸). Amino-phenolic ligands have versatile applications in inorganic as well as analytical chemistry. The flexible C—N bond in these ligands offers a tractable three-dimensional structure when coordinated to different metal centres (Riisiö *et al.*, 2012 ▸). This provides numerous applications, particularly in enzyme mimicking and catalysis, as well as extraction of trace metals (Maurya *et al.*, 2015 ▸; Riisiö *et al.*, 2013 ▸; Lee *et al.*, 2010 ▸). In the present study, we wanted to prepare a tripodal amino (bis­) phenolate Mannich base derived from 3-propanol-1-amine, formaldehyde and 2,4-di­chloro­phenol. The reaction was performed following conventional bench-top techniques by heating a solution of the reactants in methanol (Sopo *et al.*, 2006 ▸). However, probably because of the poor solubility, the dipodal product precipitated out from the incomplete reaction mixture. The dipodal product, 2,4-di­chloro-6-{[(3-hy­droxy­prop­yl)azaniumyl]­meth­yl}phenolate is stabilized by extensive intra- as well as inter­molecular hydrogen bonding and thus exists as a zwitterion. The zwitterion co-crystallized with the unreacted phenol, resulting in the serendipitous isolation of the title compound.
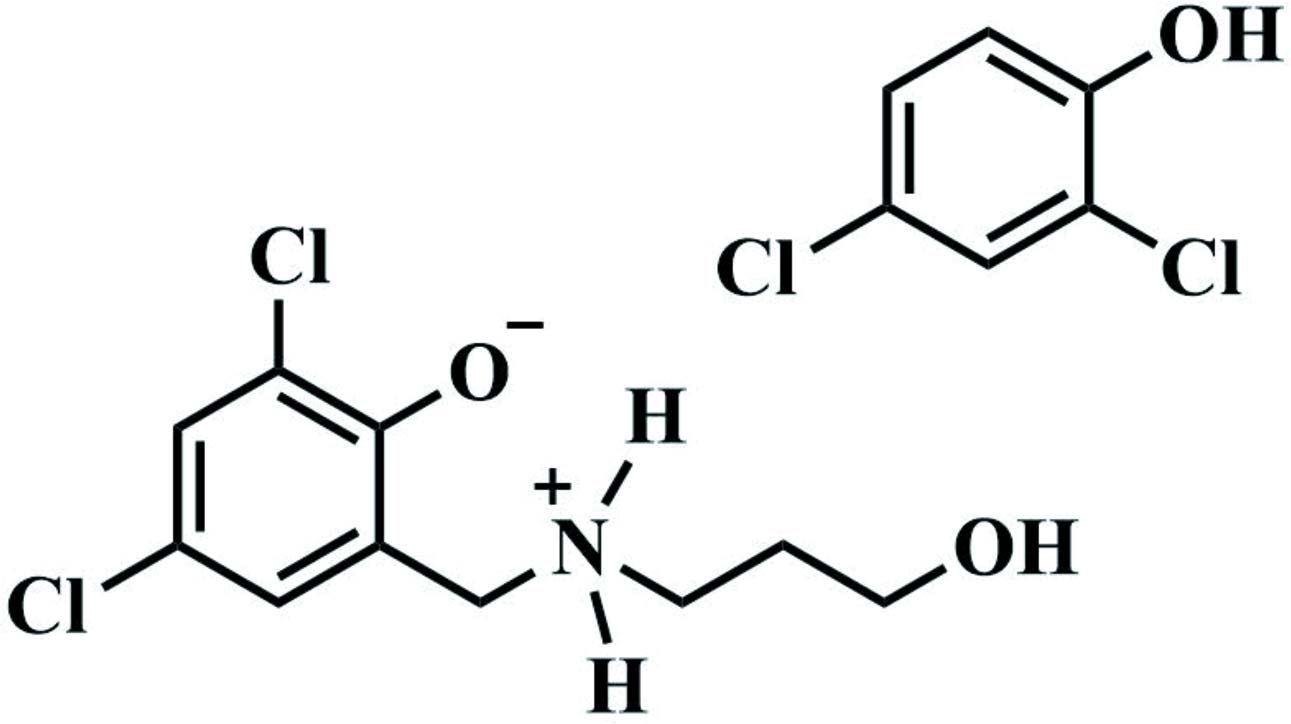


## Structural commentary   

**Figure 1:**
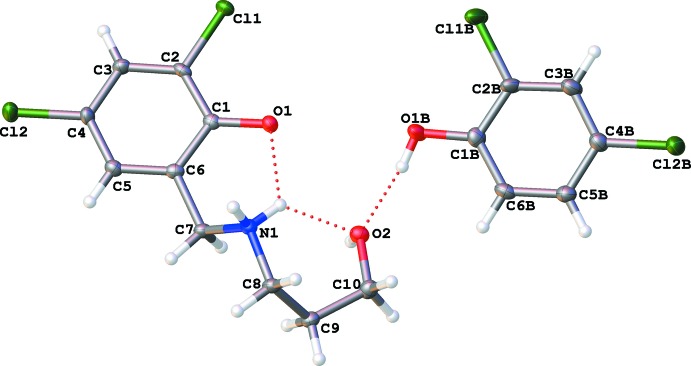
Mol­ecular structure of the title compound showing the numbering scheme and related hydrogen-bonding inter­actions. Displacement ellipsoids are shown at the 50% probability level and hydrogen bonds are drawn with red dotted lines.

The title compound crystallizes in the monoclinic crystal system, in the space group *Cc*. The mol­ecular structure of the title compound is shown in Fig. 1 ▸, and the asymmetric unit comprises a mol­ecule of both 2,4-di­chloro­phenol and 2,4-di­chloro-6-{[(3-hy­droxy­prop­yl)azaniumyl]­meth­yl}phenolate, held together by hydrogen bonds.

A complete geometrical analysis using the *Mogul* geometry check tool (Bruno *et al.*, 2004 ▸) within *Mercury* (Macrae *et al.*, 2008 ▸) did not show any unusual bond lengths or bond angles. The torsion angles of the complete azaniumyl­phenolate chain (specifically C7–N1–C8–C9–C10–O2) deviate significantly from planarity (Table 1 ▸). This can be attributed to the hydrogen bonds in this environment (see below).

The organic Mannich base exists as a zwitterion with the negative charge of phenolate being stabilized by the positively charged ammonium moiety. This is corroborated by the fact that the phenolic oxygen–carbon bond is slightly shorter [O1—C1 = 1.322 (4) Å] than the corresponding bond in the free 2,4-di­chloro­phenol [O1*B*—C1*B* = 1.355 (4) Å], indicating partial double-bond character and the presence of a phenoxide moiety in the zwitterion fragment. The ammonium nitro­gen atom adopts a slightly distorted tetra­hedral geometry.

## Supra­molecular features   

**Figure 2:**
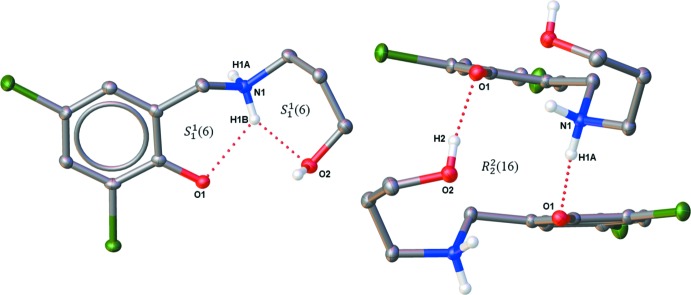
Mol­ecular structure of the zwitterionic part of the co-crystal depicting the selected hydrogen-bonding graph sets. H atoms not involved in hydrogen bonding have been omitted for clarity and hydrogen bonds are drawn with red dashed lines.

The co-crystal structure displays an extensive hydrogen-bonding network (Table 2 ▸). The zwitterion, 2,4-di­chloro-6-{[(3-hy­droxy­prop­yl)azaniumyl]­meth­yl}phenolate, is involved in intra– as well as inter­molecular hydrogen bonding. The ammonium hydrogen H1*B* takes part in a bifurcated intra­molecular hydrogen bond, N1—H1*B*⋯O1 and N1—H1*B*⋯O2, forcing the propyl chain of the zwitterion to adopt a distorted *gauche* conformation with an N1—C8—C9—C10 torsion angle of 64.5 (3)°. The other ammonium hydrogen, H1*A*, is involved in inter­molecular hydrogen bonding with the negatively charged O atom of an adjacent zwitterion [*d*(H1*A*⋯O1) = 1.76 Å], extending the hydrogen bonding into an infinite network. The two components of the co-crystal are also bonded together by inter­molecular hydrogen bonds between the phenolic proton of 2,4-di­chloro­phenol and the alcoholic oxygen of the zwitterion [*d*(H1*BA*⋯O2) = 1.82 Å]. These hydrogen bonds give rise to inter­esting graph-set patterns, which are depicted in Fig. 2 ▸. The two intra­molecular self-motifs of 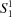
(6) are generated as a result of the bifurcation involving H1*B*, while the zwitterion inter­acts with a second zwitterion generating a large ring motif with the graph-set 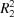
(16).

**Figure 3:**
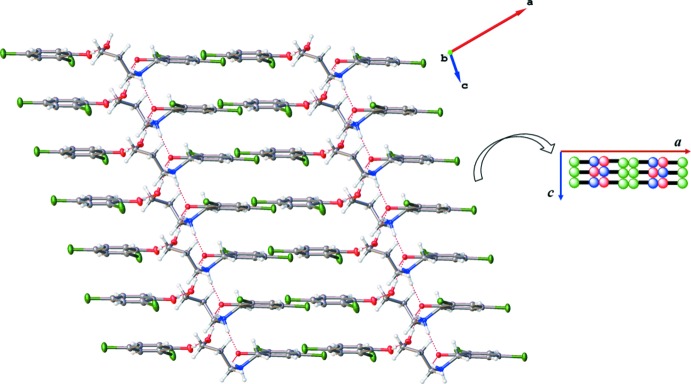
Packing diagram of the title compound viewed down the *b* axis, clearly showing the hydrogen-bonded polar channels and the 2,4-di­chloro­phenol hydro­phobic layers.

**Figure 4:**
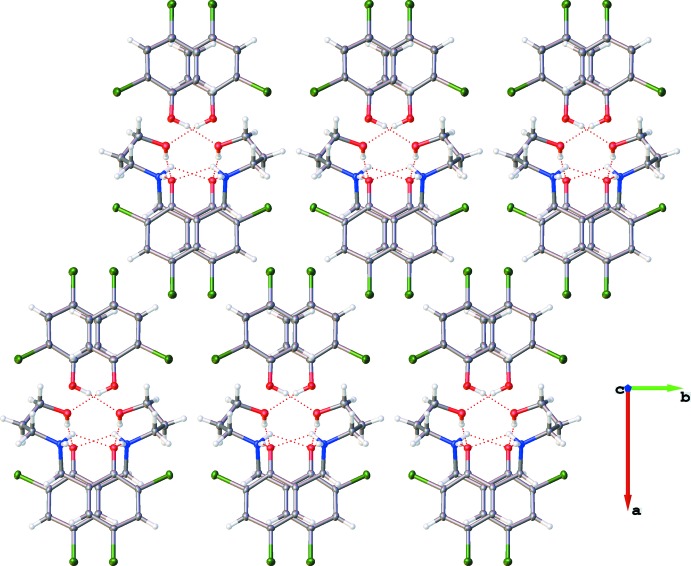
Packing diagram of the title compound viewed down the *c* axis, depicting an apparent regular mirror image resulting from the glide plane in the *ac* plane.

**Figure 5:**
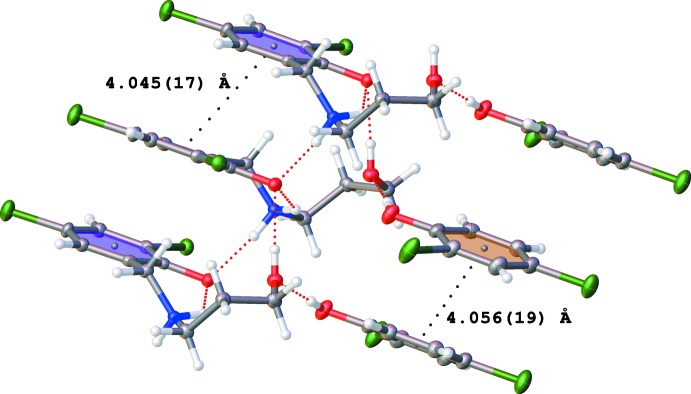
Partial packing arrangement of the title compound showing the π–π inter­action between the 2,4-di­chloro­phenol hydro­phobic layers.

The packing arrangement of the co-crystal involves alternating hydro­phobic layers of the aromatic di­chloro­phenol rings and the hydrogen-bonded polar channels. These layers stack one over the other along the *c-*axis direction and also propagate along the *a-*axis direction, thereby resulting in a ladder-like structure network (Figs. 3 ▸ and 4 ▸). The presence of the glide plane in the *ac* plane of the crystal causes the packing to appear like a regular mirror image (Fig. 4 ▸). As a result of the nature of the packing arrangement in the crystal structure, it was possible to measure the ring centroid to ring centroid distance between the di­chloro­phenol rings of the adjacent layers; this distance was found to be in the range 4.045 (17)–4.056 (19) Å (Fig. 5 ▸). The layers are stacked in this manner as a result of extensive π–π inter­actions between the phenyl rings. A detailed list of the relevant π–π inter­actions is given in Table 3 ▸.

## Database survey   

A search of the Cambridge structural database (Version 5.40, February 2019 updates; Groom *et al.*, 2016 ▸) for the zwitterionic Mannich base, 2,4-di­chloro-6-{[(3-hy­droxy­prop­yl)azaniumyl]­meth­yl}phenolate gave no hits. Search parameters that included 2,4-di­chloro­phenol and other relevant starting materials as well as the co-crystal resulted in only four hits, with one being the crystal structure of 2,4-di­chloro­phenol itself (DCPHOM; Bavoux & Perrin, 1979 ▸); the second hit was a clathrate containing 2,4-di­chloro­phenol as a guest mol­ecule within the cavities of zinc tetra­phenyl­porphyrin mol­ecules (JIVNOR; Byrn *et al.*, 1991 ▸), and the third and fourth hits were found to be two three–component co-crystal solvates [EVEYUB (Cai *et al.*, 2016 ▸) and ZISJUI (Cai & Jin, 2014 ▸)] containing H-atom-bridged 2,4-di­chloro­phenolate/2,4-di­chloro­phenol units held together by O—H⋯N and O—H⋯O hydrogen bonds. Of all the hits found in the CSD, none of the structures is reported to have any π–π inter­actions between the phenyl rings, whereas the title compound has these types of inter­actions. However, a database search for the alcoholamine fragment, NH_2_(+)–(CH_2_)_3_–OH, gave seven hits. Two of these, GIPHIX (Büttner *et al.*, 2007 ▸) and EPANUF (Pestov *et al.*, 2010 ▸), also involved intra­molecular hydrogen bonding resulting in 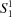
(6) graph sets, as also seen in the title compound.

## Synthesis and crystallization   

The starting materials, comprising of 3-amino­propan-1-ol, formaldehyde and 2,4-di­chloro­phenol were purchased from Sigma Aldrich and used as received without any purification. To a methano­lic solution of 3-amino-1-propanol (5 mmol, 0.38 g) was added a solution of formaldehyde (10 mmol, 0.81 g) in methanol under stirring. A solution of 2,4-di­chloro­phenol (10 mmol, 1.63 g) in methanol was added to the above mixture to afford a clear solution. The resulting solution was stirred at room temperature for two days to yield an oily solution. The oil was dissolved in diethyl ether and a few drops of methanol were added to the solution. The solution was then cooled in a refrigerator to obtain diffraction-quality single crystals.

## Refinement   

Crystal data, data collection and structure refinement details are summarized in Table 4 ▸. All carbon-bound H atoms were placed in calculated positions and refined using a riding-model approximation, with C—H = 0.95–1.00 Å and *U*_iso_(H) = 1.2*U*_eq_(C). H atoms bonded to N or O atoms were located from difference-Fourier electron-density maps and were also refined using a riding-model approximation with N—H bond distances of 0.89–0.91 Å and O—H = 0.84 Å with *U*_iso_(H) = 1.5*U*_eq_(N,O). The atom H1*A* was restrained by DFIX in *SHELX* to be at a distance of 0.88 (2) Å from N1 and by the SADI command to be equidistant from C7 and C8 (σ = 0.02 Å), so as to inhibit too much movement of this H atom during the refinement. The structure was also refined as an inversion twin, but low coverage of Friedel pairs in the data precludes the reliable determination of the absolute structure. All related structure and refinement checks were carried out with *PLATON* (Spek, 2009 ▸).

## Supplementary Material

Crystal structure: contains datablock(s) I. DOI: 10.1107/S2056989019012544/fy2140sup1.cif

Structure factors: contains datablock(s) I. DOI: 10.1107/S2056989019012544/fy2140Isup2.hkl

Supporting information file. DOI: 10.1107/S2056989019012544/fy2140Isup3.mol

CCDC reference: 1952329

Additional supporting information:  crystallographic information; 3D view; checkCIF report

## Figures and Tables

**Table 1:** Selected torsion angles (°)

C7—N1—C8—C9	69.9 (3)	C8—C9—C10—O2	−65.1 (3)
N1—C8—C9—C10	64.5 (3)		

**Table 2:** Hydrogen-bond geometry (Å, °)

*D*—H⋯*A*	*D*—H	H⋯*A*	*D*⋯*A*	*D*—H⋯*A*
N1—H1*A*⋯O1^i^	0.91	1.76	2.663 (3)	171
N1—H1*B*⋯O1	0.91	2.17	2.779 (3)	124
N1—H1*B*⋯O2	0.91	2.29	2.947 (3)	129
O2—H2⋯O1^ii^	0.84	1.80	2.634 (3)	171
O1*B*—H1*BA*⋯O2	0.84	1.82	2.653 (3)	172

Symmetry codes: (i) 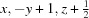; (ii) 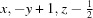.

**Table 3:** π–π inter­actions (Å, °) *Cg*1 and *Cg*2 are the centroids of the C1–C6 and C1*B*–C6*B* rings, respectively.

*Cg*⋯*Cg*	Distance	Slippage
*Cg*1⋯*Cg*1^i^	4.0449 (17)	2.006
*Cg*1⋯*Cg*1^ii^	4.0448 (17)	2.583
*Cg*2⋯*Cg*2^i^	4.0559 (19)	2.714
*Cg*2⋯*Cg*2^ii^	4.0559 (19)	1.849

Symmetry codes: (i) *x*, −*y* + 1, *z* + 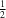; (ii) *x*, −*y* + 1, *z* − 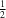.

**Table 4:** Experimental details

Crystal data	
Chemical formula	C_10_H_13_Cl_2_NO_2_·C_6_H_4_Cl_2_O
*M*_r_	413.10
Crystal system, space group	Monoclinic, *C* *c*
Temperature (K)	100
*a*, *b*, *c* (Å)	26.406 (2), 9.5558 (9), 7.1019 (6)
β (°)	101.076 (2)
*V* (Å^3^)	1758.6 (3)
*Z*	4
Radiation type	Mo *K*α
μ (mm^−1^)	0.69
Crystal size (mm)	0.39 × 0.20 × 0.17

Data collection	
Diffractometer	Bruker APEXII CCD
Absorption correction	Multi-scan (*SADABS*; Bruker, 2016 ▸)
*T*_min_, *T*_max_	0.688, 0.746
No. of measured, independent and observed [*I* > 2σ(*I*)] reflections	15694, 4227, 3842
*R*_int_	0.053
(sin θ/λ)_max_ (Å^−1^)	0.673

Refinement	
*R*[*F* ^2^ > 2σ(*F* ^2^)], *wR*(*F* ^2^), *S*	0.037, 0.092, 1.03
No. of reflections	4227
No. of parameters	220
No. of restraints	4
H-atom treatment	H-atom parameters constrained
Δρ_max_, Δρ_min_ (e Å^−3^)	0.52, −0.26
Absolute structure	Refined as an inversion twin
Absolute structure parameter	0.03 (8)

Computer programs: *APEX2* (Bruker, 2014 ▸), *SAINT* (Bruker, 2016 ▸), *SHELXT2014/5* (Sheldrick, 2015*b* ▸), *SHELXL* (Sheldrick, 2015*a* ▸) and *OLEX2* (Dolomanov *et al.*, 2009 ▸).

## supplementary crystallographic information

### Crystal data

**Table:**

C_10_H_13_Cl_2_NO_2_·C_6_H_4_Cl_2_O	*F*(000) = 848
*M**_r_* = 413.10	*D*_x_ = 1.560 Mg m^−^^3^
Monoclinic, *C**c*	Mo *K*α radiation, λ = 0.71073 Å
*a* = 26.406 (2) Å	Cell parameters from 6679 reflections
*b* = 9.5558 (9) Å	θ = 2.3–28.6°
*c* = 7.1019 (6) Å	µ = 0.69 mm^−^^1^
β = 101.076 (2)°	*T* = 100 K
*V* = 1758.6 (3) Å^3^	Plank, colourless
*Z* = 4	0.39 × 0.20 × 0.17 mm

### Data collection

**Table:**

Bruker APEXII CCD diffractometer	3842 reflections with *I* > 2σ(*I*)
φ and ω scans	*R*_int_ = 0.053
Absorption correction: multi-scan (SADABS; Bruker, 2016)	θ_max_ = 28.6°, θ_min_ = 1.6°
*T*_min_ = 0.688, *T*_max_ = 0.746	*h* = −34→35
15694 measured reflections	*k* = −12→12
4227 independent reflections	*l* = −9→9

### Refinement

**Table:**

Refinement on *F*^2^	Hydrogen site location: inferred from neighbouring sites
Least-squares matrix: full	H-atom parameters constrained
*R*[*F*^2^ > 2σ(*F*^2^)] = 0.037	*w* = 1/[σ^2^(*F*_o_^2^) + (0.051*P*)^2^] where *P* = (*F*_o_^2^ + 2*F*_c_^2^)/3
*wR*(*F*^2^) = 0.092	(Δ/σ)_max_ = 0.001
*S* = 1.03	Δρ_max_ = 0.52 e Å^−^^3^
4227 reflections	Δρ_min_ = −0.25 e Å^−^^3^
220 parameters	Absolute structure: Refined as an inversion twin
4 restraints	Absolute structure parameter: 0.03 (8)
Primary atom site location: dual	

### Special details

**Table:**

Geometry. All esds (except the esd in the dihedral angle between two l.s. planes) are estimated using the full covariance matrix. The cell esds are taken into account individually in the estimation of esds in distances, angles and torsion angles; correlations between esds in cell parameters are only used when they are defined by crystal symmetry. An approximate (isotropic) treatment of cell esds is used for estimating esds involving l.s. planes.
Refinement. Refined as a 2-component inversion twin.

### Fractional atomic coordinates and isotropic or equivalent isotropic displacement parameters (Å^2^)

**Table:**

	*x*	*y*	*z*	*U*_iso_*/*U*_eq_	
C1	0.60636 (12)	0.3974 (3)	0.4792 (4)	0.0142 (6)	
C2	0.63604 (11)	0.2750 (3)	0.5205 (4)	0.0157 (6)	
C3	0.68630 (12)	0.2726 (3)	0.6225 (4)	0.0165 (6)	
H3	0.704950	0.187257	0.645494	0.020*	
C4	0.70848 (12)	0.3991 (3)	0.6902 (5)	0.0168 (6)	
C5	0.68172 (11)	0.5238 (3)	0.6521 (4)	0.0165 (6)	
H5	0.697687	0.609545	0.698121	0.020*	
C6	0.63169 (11)	0.5240 (3)	0.5469 (4)	0.0147 (6)	
O1	0.55807 (8)	0.3971 (2)	0.3846 (3)	0.0155 (5)	
C7	0.60594 (11)	0.6628 (3)	0.4870 (4)	0.0158 (5)	
H7A	0.625976	0.738673	0.561830	0.019*	
H7B	0.606421	0.679235	0.349730	0.019*	
N1	0.55136 (9)	0.6690 (2)	0.5164 (3)	0.0147 (4)	
H1A	0.550581	0.642000	0.638674	0.018*	
H1B	0.532185	0.606588	0.435781	0.018*	
C8	0.52697 (11)	0.8107 (3)	0.4826 (4)	0.0175 (5)	
H8A	0.550100	0.879869	0.559396	0.021*	
H8B	0.494264	0.809926	0.531229	0.021*	
C9	0.51540 (11)	0.8601 (3)	0.2744 (4)	0.0175 (6)	
H9A	0.503078	0.958053	0.271129	0.021*	
H9B	0.547924	0.859396	0.224456	0.021*	
C10	0.47556 (12)	0.7729 (3)	0.1416 (4)	0.0202 (6)	
H10A	0.443328	0.769495	0.193267	0.024*	
H10B	0.467599	0.817985	0.013813	0.024*	
O2	0.49333 (8)	0.6335 (2)	0.1213 (3)	0.0184 (4)	
H2	0.513930	0.633301	0.044658	0.028*	
Cl1	0.60850 (3)	0.11500 (7)	0.43483 (9)	0.01842 (17)	
Cl2	0.77111 (3)	0.39949 (8)	0.82500 (10)	0.0250 (2)	
O1B	0.44186 (9)	0.3939 (2)	0.1038 (4)	0.0241 (6)	
H1BA	0.455585	0.473101	0.102256	0.036*	
C1B	0.39283 (14)	0.3987 (3)	0.0027 (5)	0.0199 (7)	
C2B	0.36413 (13)	0.2748 (4)	−0.0255 (5)	0.0222 (7)	
C3B	0.31382 (13)	0.2721 (4)	−0.1258 (5)	0.0248 (7)	
H3B	0.295037	0.186795	−0.143940	0.030*	
C4B	0.29142 (13)	0.3965 (3)	−0.1992 (5)	0.0218 (7)	
C5B	0.31829 (12)	0.5215 (4)	−0.1709 (4)	0.0216 (7)	
H5B	0.302285	0.606604	−0.219504	0.026*	
C6B	0.36880 (12)	0.5211 (3)	−0.0710 (4)	0.0205 (6)	
H6B	0.387375	0.606724	−0.052605	0.025*	
Cl2B	0.22825 (3)	0.39666 (9)	−0.32799 (12)	0.0308 (2)	
Cl1B	0.39215 (3)	0.11872 (9)	0.07078 (15)	0.0370 (3)	

### Atomic displacement parameters (Å^2^)

**Table:**

	*U*^11^	*U*^22^	*U*^33^	*U*^12^	*U*^13^	*U*^23^
C1	0.0169 (16)	0.0145 (16)	0.0124 (14)	−0.0011 (11)	0.0055 (11)	−0.0008 (9)
C2	0.0192 (15)	0.0122 (15)	0.0165 (13)	−0.0052 (12)	0.0057 (11)	−0.0028 (10)
C3	0.0178 (15)	0.0129 (16)	0.0189 (13)	0.0016 (12)	0.0036 (11)	−0.0006 (10)
C4	0.0110 (15)	0.0175 (17)	0.0210 (15)	−0.0005 (11)	0.0011 (12)	−0.0027 (11)
C5	0.0172 (15)	0.0150 (16)	0.0177 (14)	−0.0015 (12)	0.0048 (11)	−0.0019 (10)
C6	0.0178 (14)	0.0128 (15)	0.0151 (13)	0.0005 (12)	0.0069 (10)	−0.0004 (10)
O1	0.0145 (12)	0.0156 (12)	0.0163 (10)	−0.0013 (8)	0.0029 (8)	−0.0017 (7)
C7	0.0168 (13)	0.0120 (14)	0.0193 (13)	0.0005 (10)	0.0049 (10)	0.0008 (9)
N1	0.0168 (11)	0.0144 (11)	0.0130 (11)	−0.0011 (9)	0.0033 (8)	0.0003 (8)
C8	0.0223 (14)	0.0120 (13)	0.0182 (13)	0.0004 (10)	0.0038 (10)	0.0005 (10)
C9	0.0199 (14)	0.0154 (14)	0.0174 (14)	0.0010 (10)	0.0037 (11)	0.0026 (10)
C10	0.0212 (14)	0.0191 (15)	0.0197 (14)	0.0031 (11)	0.0026 (10)	−0.0015 (11)
O2	0.0193 (10)	0.0168 (10)	0.0201 (10)	−0.0005 (8)	0.0060 (8)	−0.0012 (7)
Cl1	0.0201 (4)	0.0128 (4)	0.0216 (3)	−0.0027 (3)	0.0023 (3)	−0.0022 (3)
Cl2	0.0169 (4)	0.0194 (4)	0.0353 (5)	0.0010 (3)	−0.0038 (3)	−0.0041 (3)
O1B	0.0159 (13)	0.0151 (13)	0.0403 (15)	−0.0022 (8)	0.0027 (10)	−0.0003 (9)
C1B	0.0152 (16)	0.0220 (19)	0.0235 (17)	−0.0002 (12)	0.0062 (12)	−0.0015 (11)
C2B	0.0199 (16)	0.0120 (17)	0.0352 (17)	0.0023 (13)	0.0068 (13)	0.0024 (12)
C3B	0.0211 (18)	0.0162 (19)	0.0373 (18)	−0.0030 (13)	0.0059 (14)	−0.0017 (13)
C4B	0.0184 (18)	0.0197 (18)	0.0274 (17)	−0.0020 (12)	0.0046 (14)	−0.0020 (12)
C5B	0.0234 (17)	0.0164 (17)	0.0251 (16)	−0.0007 (13)	0.0045 (12)	0.0015 (12)
C6B	0.0243 (17)	0.0142 (16)	0.0242 (16)	−0.0043 (13)	0.0076 (12)	−0.0022 (11)
Cl2B	0.0200 (5)	0.0207 (5)	0.0474 (6)	−0.0024 (3)	−0.0038 (4)	0.0031 (4)
Cl1B	0.0209 (5)	0.0167 (5)	0.0705 (7)	0.0004 (3)	0.0018 (5)	0.0095 (4)

### Geometric parameters (Å, º)

**Table:**

C1—C2	1.407 (4)	C9—H9A	0.9900
C1—C6	1.421 (4)	C9—H9B	0.9900
C1—O1	1.322 (4)	C9—C10	1.520 (4)
C2—C3	1.385 (4)	C10—H10A	0.9900
C2—Cl1	1.751 (3)	C10—H10B	0.9900
C3—H3	0.9500	C10—O2	1.429 (3)
C3—C4	1.388 (4)	O2—H2	0.8400
C4—C5	1.386 (4)	O1B—H1BA	0.8400
C4—Cl2	1.744 (3)	O1B—C1B	1.355 (4)
C5—H5	0.9500	C1B—C2B	1.400 (5)
C5—C6	1.387 (4)	C1B—C6B	1.384 (5)
C6—C7	1.514 (4)	C2B—C3B	1.382 (5)
C7—H7A	0.9900	C2B—Cl1B	1.745 (3)
C7—H7B	0.9900	C3B—H3B	0.9500
C7—N1	1.496 (3)	C3B—C4B	1.384 (5)
N1—H1A	0.9100	C4B—C5B	1.385 (4)
N1—H1B	0.9100	C4B—Cl2B	1.742 (4)
N1—C8	1.499 (3)	C5B—H5B	0.9500
C8—H8A	0.9900	C5B—C6B	1.385 (4)
C8—H8B	0.9900	C6B—H6B	0.9500
C8—C9	1.526 (4)		
			
C2—C1—C6	115.5 (3)	C9—C8—H8A	108.3
O1—C1—C2	123.3 (3)	C9—C8—H8B	108.3
O1—C1—C6	121.3 (3)	C8—C9—H9A	108.6
C1—C2—Cl1	118.4 (2)	C8—C9—H9B	108.6
C3—C2—C1	124.2 (3)	H9A—C9—H9B	107.6
C3—C2—Cl1	117.3 (2)	C10—C9—C8	114.7 (2)
C2—C3—H3	121.2	C10—C9—H9A	108.6
C2—C3—C4	117.7 (3)	C10—C9—H9B	108.6
C4—C3—H3	121.2	C9—C10—H10A	109.2
C3—C4—Cl2	119.0 (2)	C9—C10—H10B	109.2
C5—C4—C3	121.1 (3)	H10A—C10—H10B	107.9
C5—C4—Cl2	120.0 (2)	O2—C10—C9	111.8 (2)
C4—C5—H5	119.9	O2—C10—H10A	109.2
C4—C5—C6	120.2 (3)	O2—C10—H10B	109.2
C6—C5—H5	119.9	C10—O2—H2	109.5
C1—C6—C7	119.6 (3)	C1B—O1B—H1BA	109.5
C5—C6—C1	121.3 (3)	O1B—C1B—C2B	118.9 (3)
C5—C6—C7	118.8 (3)	O1B—C1B—C6B	123.4 (3)
C6—C7—H7A	109.0	C6B—C1B—C2B	117.7 (3)
C6—C7—H7B	109.0	C1B—C2B—Cl1B	119.3 (3)
H7A—C7—H7B	107.8	C3B—C2B—C1B	122.0 (3)
N1—C7—C6	112.8 (2)	C3B—C2B—Cl1B	118.6 (3)
N1—C7—H7A	109.0	C2B—C3B—H3B	120.8
N1—C7—H7B	109.0	C2B—C3B—C4B	118.5 (3)
C7—N1—H1A	108.7	C4B—C3B—H3B	120.8
C7—N1—H1B	108.7	C3B—C4B—C5B	121.1 (3)
C7—N1—C8	114.1 (2)	C3B—C4B—Cl2B	119.7 (3)
H1A—N1—H1B	107.6	C5B—C4B—Cl2B	119.2 (3)
C8—N1—H1A	108.7	C4B—C5B—H5B	120.4
C8—N1—H1B	108.7	C4B—C5B—C6B	119.2 (3)
N1—C8—H8A	108.3	C6B—C5B—H5B	120.4
N1—C8—H8B	108.3	C1B—C6B—C5B	121.5 (3)
N1—C8—C9	115.8 (2)	C1B—C6B—H6B	119.3
H8A—C8—H8B	107.4	C5B—C6B—H6B	119.3
			
C1—C2—C3—C4	0.8 (4)	N1—C8—C9—C10	64.5 (3)
C1—C6—C7—N1	50.1 (3)	C8—C9—C10—O2	−65.1 (3)
C2—C1—C6—C5	−2.1 (4)	Cl1—C2—C3—C4	179.7 (2)
C2—C1—C6—C7	171.6 (2)	Cl2—C4—C5—C6	−179.2 (2)
C2—C3—C4—C5	−1.8 (5)	O1B—C1B—C2B—C3B	179.5 (3)
C2—C3—C4—Cl2	178.3 (2)	O1B—C1B—C2B—Cl1B	0.3 (4)
C3—C4—C5—C6	0.8 (5)	O1B—C1B—C6B—C5B	−179.0 (3)
C4—C5—C6—C1	1.2 (4)	C1B—C2B—C3B—C4B	−0.3 (5)
C4—C5—C6—C7	−172.6 (3)	C2B—C1B—C6B—C5B	−0.6 (5)
C5—C6—C7—N1	−136.0 (3)	C2B—C3B—C4B—C5B	−0.9 (5)
C6—C1—C2—C3	1.2 (4)	C2B—C3B—C4B—Cl2B	179.5 (2)
C6—C1—C2—Cl1	−177.8 (2)	C3B—C4B—C5B—C6B	1.4 (5)
C6—C7—N1—C8	172.5 (2)	C4B—C5B—C6B—C1B	−0.6 (5)
O1—C1—C2—C3	−178.7 (3)	C6B—C1B—C2B—C3B	1.1 (5)
O1—C1—C2—Cl1	2.4 (4)	C6B—C1B—C2B—Cl1B	−178.2 (2)
O1—C1—C6—C5	177.7 (3)	Cl2B—C4B—C5B—C6B	−179.0 (2)
O1—C1—C6—C7	−8.6 (4)	Cl1B—C2B—C3B—C4B	178.9 (3)
C7—N1—C8—C9	69.9 (3)		

### Hydrogen-bond geometry (Å, º)

**Table:**

*D*—H···*A*	*D*—H	H···*A*	*D*···*A*	*D*—H···*A*
N1—H1*A*···O1^i^	0.91	1.76	2.663 (3)	171
N1—H1*B*···O1	0.91	2.17	2.779 (3)	124
N1—H1*B*···O2	0.91	2.29	2.947 (3)	129
O2—H2···O1^ii^	0.84	1.80	2.634 (3)	171
O1*B*—H1*BA*···O2	0.84	1.82	2.653 (3)	172

Symmetry codes: (i) *x*, −*y*+1, *z*+1/2; (ii) *x*, −*y*+1, *z*−1/2.
